# Aged care clinical mentoring model of change in nursing homes in China: study protocol for a cluster randomized controlled trial

**DOI:** 10.1186/s12913-018-3596-6

**Published:** 2018-10-25

**Authors:** Hui Feng, Hui Li, Lily Dongxia Xiao, Shahid Ullah, Pan Mao, Yunxia Yang, Hengyu Hu, Yinan Zhao

**Affiliations:** 1 Xiangya school of nursing, Central South University, Changsha, Hunan province China; 2 College of Nursing and Health Sciences, Flinders University, GPO Box 2100, Adelaide, SA 5001 Australia; 3 South Australian Health and Medical Research Institute, Adelaide, South Australia Australia; 4 Henan Provincial Peoples Hospital, Zhengzhou, Henan Province China

**Keywords:** Cluster randomized controlled trial, Mentoring, Nursing home, Quality of care, Quality improvement

## Abstract

**Background:**

Residents living in nursing homes usually have complex healthcare needs and require a comprehensive care approach to identifying and meeting their care needs. Suboptimal quality of care is reported in nursing homes and is associated with the poor health and well-being of the residents, the burden on acute care hospitals and the high costs of healthcare for the government.

The aim of this study is to test the hypothesis that an Aged Care Clinical Mentoring Model will create and sustain evidence-based quality improvement in priority areas and will be cost-effective in nursing homes in Hunan Province, China.

**Methods:**

A cluster randomized controlled trial will be applied to the study. Fourteen nursing homes will be randomly allocated to either the intervention group (*n* = 7) or the control group (*n* = 7). Forty staff will be recruited from each nursing home and the estimated sample size will be 280 staff in each group. The intervention includes a structured, evidence-based quality improvement education program for staff to facilitate knowledge translation in evidence-based quality improvement targeting urinary incontinence, pressure injury and falls prevention. The primary outcomes are nursing homes’ capacity to create and sustain quality improvement, staff perceptions of person-centered care, self-reported quality of care by residents and selected quality indicators at 12 months follow-up adjusted for baseline value. Secondary outcomes are residents’ quality of life, residents’ unplanned admissions to acute care hospitals, quality of care reported by staff, staff job satisfaction and staff intention to leave adjusted for baseline value. A mixed linear regression model will be adopted to compare the significant differences between groups over a 12-month period.

**Discussion:**

Although the Aged Care Clinical Mentoring Model has been tested as an effective model to bring positive changes in nursing homes in a high-income country, factors affecting the adaptation of the model in nursing homes in low- and middle-income countries are unknown. The carefully planned intervention protocol enables the project team to consider enablers and barriers when adapting the Model. Therefore, strategies and resources will be in place to manage challenges while demonstrating best practice in this study.

**Trial registration:**

Prospectively registered via Chinese Clinical Trial Registry (ChiCTR), ChiCTR-IOC-17013109, Registered on 25 October 2017.

**Electronic supplementary material:**

The online version of this article (10.1186/s12913-018-3596-6) contains supplementary material, which is available to authorized users.

## Background

The Chinese population is aging dramatically due to improved life-expectancy and a decrease in fertility rate [1]. In 2016, the number of people aged over 60 years reached 230 million [2]. It was also estimated that more than 100 million people aged 60 years or over had chronic diseases [3]. The number of older people living with disabilities reached 37 million and those living with dementia reached 9.19 million [3]. The demand for nursing home care for older people with high-dependence care needs has reached an unprecedented high in China [1]. By 2016, the number of nursing homes was 140 thousand with 7.3 million beds, and the average annual growth rate of nursing home beds was 8.6% nation-wide [4]. The majority of residents in nursing homes have dementia or cognitive impairment [5]. The rapid growth of nursing homes has raised great concern in the public about the quality of care for nursing home residents in China [6]. There are few evidence-based intervention studies to improve quality of care in nursing homes. This study will address the gap in research by adapting and evaluating an Aged Care Clinical Mentoring Model that has been tested as an effective model of change in evidence-based quality improvement in nursing homes in Australia [7].

Similar to many low- and middle-income countries, China has experienced significant social and economic transitions in the past four decades, characterized by improved employment rates for women and increased internal migration for employment or career development opportunities. This surge in employment for women has weakened the traditional family-based care for older people, [1] the demand for nursing home care for older people has increased dramatically due to the lack of family caregivers [1, 8]. However, nursing homes are currently underdeveloped to provide residents with high-quality care, evidenced by reports on unmet care needs, suboptimal care and high hospitalization rates [6].

Lack of workforce development to implement evidence-based quality of care for residents is one of the key factors amongst many that contribute to undesirable care outcomes in nursing homes [9]. Registered nurses are a minority in the workforce in nursing homes, and have limited educational preparation in leading nursing home care [10]. A large proportion of staff members in nursing homes are untrained personal care assistants (PCAs) who are mainly migrant workers from rural areas and have low levels of education with little to no training in nursing home care [10]. In addition, a low staff to resident ratio in nursing homes is widely reported and is attributed to unmet care needs for residents [10].

Quality of care is defined as “the degree to which health services for individuals and populations increase the likelihood of desired health outcomes and are consistent with current professional knowledge” [11, 12]. This definition strongly suggests that quality improvement needs to be based on current research evidence. Staff education about evidence-based practice and knowledge translation activities are two crucial components in evidence-based quality improvement [7, 13]. However, nursing homes are usually perceived as resource-poor settings for implementing evidence-based quality improvement [14]. Building nursing homes’ capacity to create and sustain quality improvement is a priority in the aged care system to ensure that quality of care is achieved [15].

The most frequently reported indicators of poor quality care in a Chinese nursing home context are falls, fall-related injuries, pressure ulcers and urinary incontinence among residents. Residents living with dementia are associated with a high risk of these poor quality care outcomes [16]. The estimated prevalence rate of urinary incontinence is around 65.8%, the fall rate is around 14.7–34% and the pressure ulcer rate is around 7.1–25% in nursing homes in China [17–19]. Prevention of these adverse events has been identified as a priority quality improvement as they have negative consequences for residents and for healthcare costs [18].

Clinical Mentor as a model of change in evidence-based quality improvements has been widely reported and recognized as a successful model globally [20, 21]. An Aged Care Clinical Mentor (ACCM) is defined as “a leader who facilitates improved quality of care for older people using best practice by providing and encouraging professional development in colleagues through communication, education and peer support” [9, 21, 22]. In the ACCM model in Australia, Registered Nurses (RNs) were prepared and supported to lead evidence-based quality improvement activities and were appointed as a clinical mentor for care staff (mentees). The ACCM model was initiated by the Australian Government and was tested in four aged care facilities and four community aged care settings across two states in Australia [9, 22]. The results revealed that the ACCM model was an effective workforce model to improve aged care organizations’ capacity to create and sustain quality improvement [9]. The aim of this study is to create and sustain quality improvement in nursing homes by implementing the ACCM model in Hunan Province, China.

## Methods

### Study design

The ACCM model will be tested using a single-blind cluster randomized controlled trial (RCT) design. The study will be undertaken in Hunan Province, the seventh largest populated province in China, and home to 12.01 million people aged 60 or over where one-sixth of them were living with disabilities in 2015 [23]. Fourteen nursing homes in Hunan Province will be randomly selected and divided into two groups: the intervention group and the control group. Cluster RCT will be applied to the study to reduce influence from the interplay of subjects from the same nursing home. Because of the nature of the intervention, it is not possible to mask the study assignment from the research interventionists (the project team who will deliver the intervention), though staff at participating sites will be unaware of group assignment. The outcomes assessment will be performed by independent investigators who will be blinded to the group assignment. The standard protocol items named “Recommendations for Interventional Trials (SPIRIT)” that provides an overview of schedule of enrolment, interventions and assessments is presented in Table 1. The SPIRIT checklist is shown in Additional file 1.

**Table 1:**
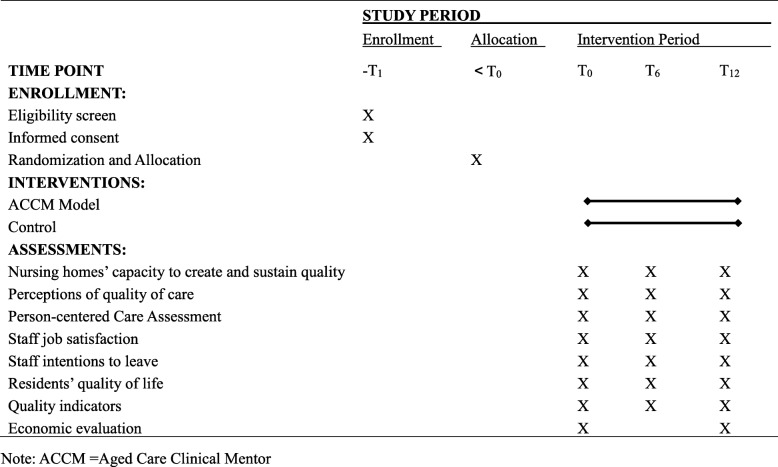
schedule of enrolment, interventions and assessments

### Hypotheses

Primary hypothesis: Nursing homes that implement the ACCM model will report enhanced capacity to create and sustain evidence-based quality (H_1_), improved staff perceptions of person-centered care (H_2_), and higher levels of residents’ or their families’ perception of quality of care (H_3_) compared to the control group.

Secondary hypothesis: Nursing homes that implement the ACCM model will report improved quality of life for residents (H_4_), reduced unplanned admissions to acute care hospitals (H_5_), higher staff perception of quality of care (H_6_), increased job satisfaction for staff (H_7_), and decreased staff intention to leave (H_8_).

### Inclusion and exclusion criteria

Nursing homes will be included in the study if: (i) they have 150 beds or more; (ii) they have RNs in each unit; (iii) they are willing to participate in the study voluntarily. Nursing homes will be excluded from the study if they are participating in other training and research activities.

### Participants and sample size

Participants will be staff who provide direct care for residents. The sample size is based on one of the primary outcomes: staff perceptions of person-centered care measured by the ‘Approaches to Dementia Questionnaire’ (ADQ). In an earlier cluster RCT to improve care quality and safety through a 12-month clinical leadership program in aged care [24], the researchers found no differences in care staff attitudes toward dementia and dementia care assessed by ADQ between the intervention and control sites at 12 months post-intervention. Therefore, in our study, we have set a clinically meaningful change of 1.0 ADQ scores and 85% power to detect a difference of 1.2 standard deviation between groups at the 5% level of significance.

Since randomization will be conducted by the nursing homes (clusters), the sample size was adjusted to take into account the design effect. We have considered a large intra-class correlation coefficient of 0.26 (average estimate from a nursing home staff training intervention carried out in England and Wales [25]) and an average cluster size of 40 nursing staff. Assuming an alpha error of 0.05 and a beta error of 20%, the required cluster number was 7 for each group (251 nursing staff per group). Allowing for an attrition rate of 10–12%, we would require around 280 staff in each arm of the project.

### Randomization

**Fig. 1:**
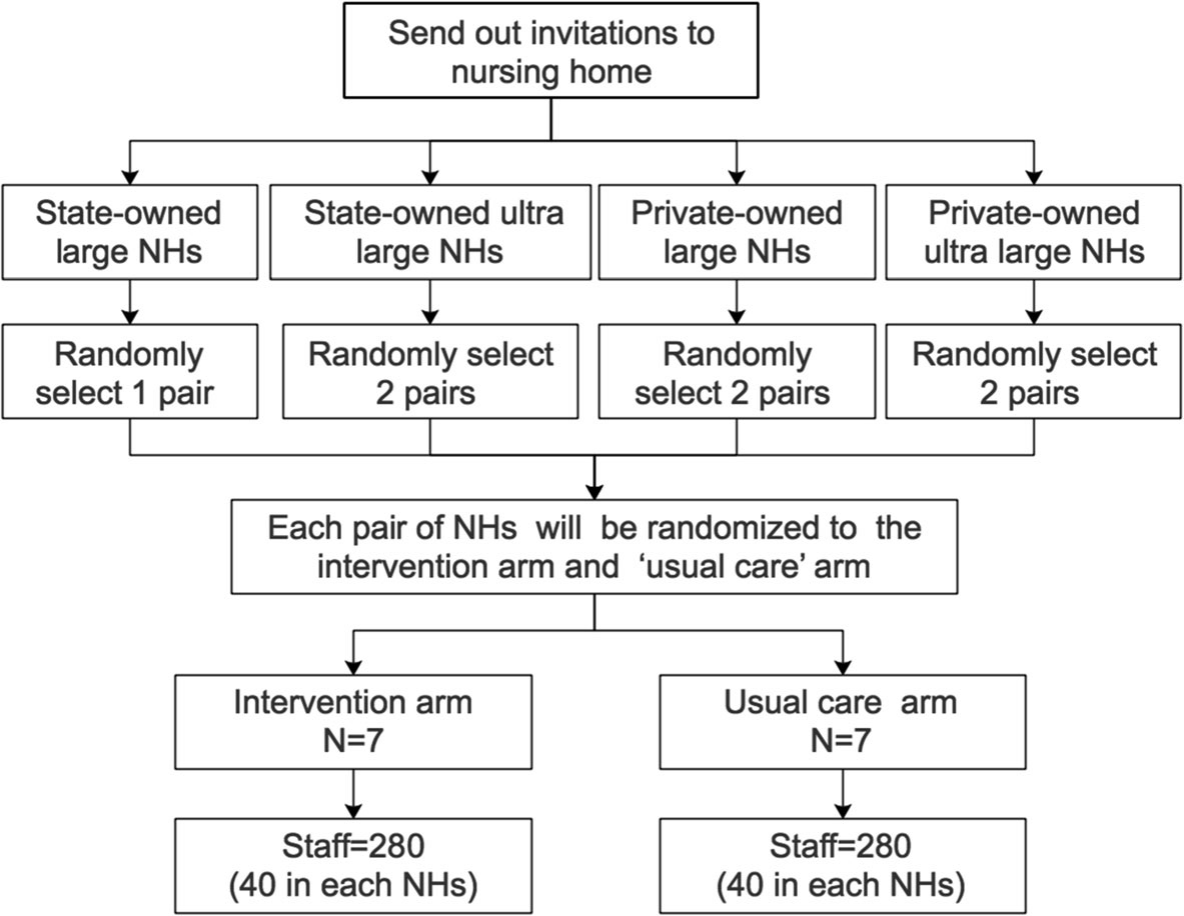
Randomization

Based on research experience, only nursing homes with 150 beds or more have RNs on each unit to manage care services and to supervise PCAs. Moreover, there are two types of nursing.

homes: state-owned nursing homes and not-for-profit privately-owned nursing homes (the majority) and the former has a greater wealth of resources. The size of nursing homes and sources of funding are major characteristics that influence quality of care [26]. To minimize difference between the two groups, participating nursing homes will be grouped into four categories according to ownership and size, and modified stratification will be applied to the group assignment to improve the equivalence of the intervention group and the control group. Computer generated random numbers will be used for group assignment and this will be performed by an independent statistician (see Fig. 1).

### Description of the ACCM model intervention

**Fig. 2:**
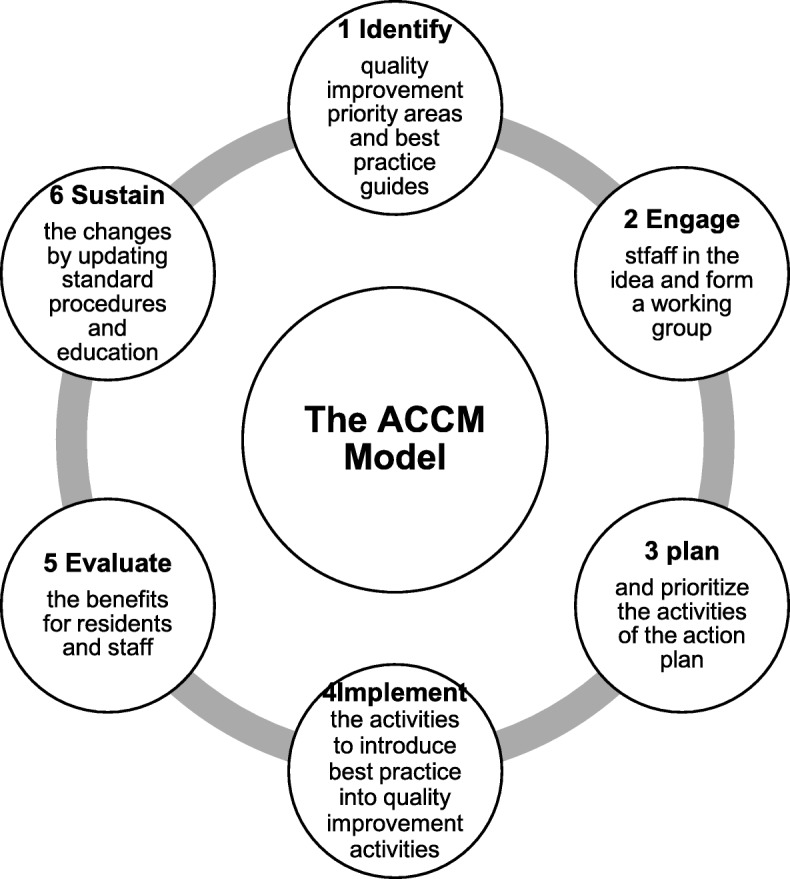
Six Steps to Better Practice (Morey et al., 2015, p.15–16)

**Fig. 3:**
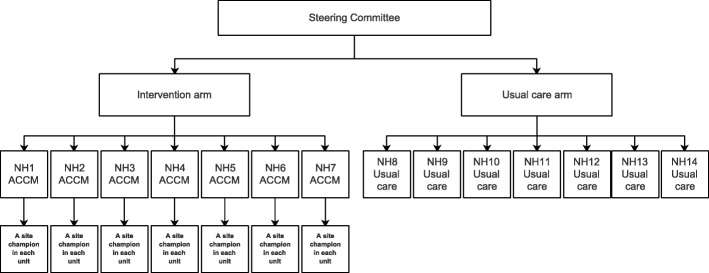
Organizational Structure of the ACCM Model

This study has adapted the key characteristics of the Australian ACCM Model of Change described as the ‘Six Steps to Better Practice’ (see Fig. 2). The organizational structure of the ACCM Model is outlined in Fig. 3. The Steering Committee will be specifically established for this trial and will comprise the Principle Investigator, Co-Principle Investigators, Hunan Government representatives, representatives from the main project participants and representatives from the seven intervened nursing homes. Each nursing home will appoint a RN as a Clinical Mentor and each unit will appoint a RN or licensed nurse as a Site Champion.

### Description of the ACCM model intervention

The intervention components, as described in the following section, will continue for 12 months. An evidence-based knowledge translation education program will be developed to enable the implementation of the ACCM model, and includes five modules: (i) Introduction to ACCM model; (ii) Leadership and clinical coaching in ACCM model; (iii) Urinary incontinence prevention; (iv) Pressure injury prevention and (v) falls prevention (see Table 2). The education program is based on the aim of the trial and the priorities in quality improvement identified by stakeholders in an industry consultation and a comprehensive literature search. The train-the-trainer method will be applied to implement the education program in which the Clinical Mentors will attend a 3-day intensive workshop (modules 1–5) and the Site Champions will attend a 2-day workshop (modules 2–5). The Clinical Mentor will work in collaboration with the Site Champions to implement the education program through education and training activities for staff. In each learning module, the project team will provide the Clinical Mentors and Site Champions with facilitator’s manuals and provide staff with a workbook to enhance interactive learning.

**Table 2:** Structured and Evidence-Based Quality Improvement Education Program

Modules	Participants
Module 1. The strategies and procedures for the Aged Care Clinical Mentor to Create and Sustain Quality Improvement	ACCMs
Module 2. Effective leadership and clinical coaching for staff	ACCMs & Site Champions
Module 3. Evidence-based urinary incontinence management for residents	ACCMs & Site Champions
Module 4. Evidence-based pressure injury prevention for residents	ACCMs & Site Champions
Module 5. Evidence-based falls prevention	ACCMs & Site Champions

Note: ACCMs = Aged Care Clinical Mentors

The Clinical Mentors will develop an action plan every three months and discuss the action plan in the regular Steering Committee meetings in order to gain feedback from the Committee to revise or amend the plan. The Clinical Mentor will also receive regular written feedback based on the analysis of outcome measures on baseline data, and at 6 and 12 months post-intervention to foster quality improvement. An Academic Coach, who is an academic member of the University and is specialized in geriatric nursing, will be appointed to support Clinical Mentors to implement the action plan, monitor the process, and update standards, procedures, and in-service education based on the outcomes of the project. The Clinical Mentors and Site Champions will be given paid time of eight hours per week to deliver education modules and mentor staff to translate knowledge at the point of care.

### Description of control group

Nursing homes in the control group will receive routine training and information about the current care standards for residents that are applicable all nursing homes in Hunan Province. If they have further questions about the quality of care for the project team, they will be referred to relevant resources provided by the Hunan Provincial Civil Affairs Department that, again, are available to all nursing homes.

### Recruitment

Recruitment of nursing homes and care staff will be based on the selection criteria. A letter of invitation will be sent to all eligible nursing home managers to gain their informed consent to participate in the trial. Managers will administer confidentiality agreements regarding group assignment with the project team. However, the ACCM model will be introduced to all participating nursing homes after the trial.

### Outcome measures and data collection

#### Primary outcomes

Primary outcomes include: (i) Nursing homes’ capacity to create and sustain quality improvement; (ii) Staff perceptions of Person-centered Care; (iii) Self-reported quality of care by residents; and (iv) quality indicators that include falls, fall-related injuries, pressure ulcers and urinary incontinence at 12 months follow-up adjusted for baseline value.

#### Secondary outcomes

Secondary outcomes are the following outcomes adjusted for baseline value: (i) Residents’ quality of life; (ii) Residents’ unplanned admissions to acute care hospitals due to adverse events, as described in the primary outcomes; (iii) Self-reported quality of care by staff; (iv) Staff job satisfaction; (v) Staff intention to leave.

#### Instruments

The instruments and their relationship to outcomes and hypotheses are presented in Table 3 [12, 27–33].

**Table 3:** Description of instruments used in outcome measures

Outcomes	Instruments description	Hypotheses
Nursing homes’ capacity to create and sustain quality improvement	Shortell’s Organization and Management Survey had been adapted to nursing home to evaluate nursing homes’ capacity to create and sustain quality improvement by Scott [27]. The scale has 35 items, consists of 5 subscales related to communication and leadership. Cronbach’s α of the Chinese version is 0.87 [27].	H_1_- nursing homes’ capacity to create and sustain evidence-based quality
Perceptions of quality of care	A single item on a 4-point Likert scale which is dichotomized as very low or rather low opposed to rather high or very high [12].	H_6_&H_3_ - perception of quality of care
Person-centered Care Assessment Tool	Person-centered Care Assessment Tool consists of three subscales: the extent of personalizing care, amount of organizational support, degree of environmental accessibility, Cronbach’s α of the Chinese version is 0.68 [28, 29].	H_2_- staff’s perceptions of Person-centered Care
Staff job satisfaction	Minnesota Satisfaction Questionnaire in short form has 20 items was used to examine satisfaction with professional life, Cronbach’s α of the Chinese version is 0.95 [30].	H_7_- job satisfaction
Staff intentions to leave	Turnover Intention Scale has 6 items and Items are scored using 4-point Likert. The Chinese version has been tested and used to assess the turnover intention of care staff. Cronbach’s α of the Chinese version is 0.92 [31].	H_8_- staff intention to leave
Residents’ quality of life	The12-item Short-Form Health Survey has 8 domains include Physical Functioning, Role-Physical, Bodily Pain, General Health, Vitality, Social Functioning, Role-Emotional, and Mental Health. Cronbach’s α of the Chinese version is 0.91 [32].	H_4_- residents’ quality of life
	Quality of Life in Late-Stage of Dementia has 3 factors and 11 items, with the factors being depressive mood, behavioral symptoms of discomfort, and positive behavioral signs of social interaction. Cronbach’s α of the Chinese version is 0.74 [33].	

#### Economic evaluation

Medical costs for each resident will be documented and collected in relation to treatment for fall-related injuries, new pressure ulcers, new urinary tract infections, and visits and readmissions to acute care hospitals. Cost-effectiveness will be expressed through a cost saving ratio and/or the outcomes per cost of intervention.

#### Data collection

The data will be collected before the intervention, and at 6 and 12 months after the commencement of intervention, for both intervention and control groups. A project manager will be appointed to coordinate the data collection process. Independent investigators who are blind to study assignment will distribute, collect and assist residents and staff to complete the survey. The Clinical Mentors in the intervention arm, and a liaison person in each control nursing home, will be tasked to work together with the project manager to complete the incident record form on falls, fall-related injuries, pressure ulcers and urinary tract infections on a weekly basis. The quality indicators will be carefully tracked by the following methods: (i) weekly follow-up and surveillance of the adverse events that will be recorded and submitted by the site managers; (ii) checking of residents’ progress notes and handover reports by research assistants to verify the incidents.

Each staff member and resident will be assigned a code number to protect their identity. This will also allow the researcher to re-identify them if needed. In order to be able to link all three surveys together without revealing staff identities, the project team will implement an identification method in which care staff members will be asked to write their maternal grandparents’ first names next to a unique site character on the front page of each survey.

### Statistical methods

Data from the trial will be analyzed using STATA software version 14.0 (Stata Corp, College Station, Texas, USA). All data will be checked for accuracy and a missing data analysis will be undertaken. The trial will adhere to the ‘CONSORT statement: extension to cluster randomized trials’ [34]. The data will be analyzed on an intention-to-treat basis, based on patient assignment.

A mixed linear regression model will be adopted to compare the significant differences between groups over a 12-month period. The mixed linear regression model will be adjusted by baseline value and potential confounding variables, for example, the sites and staff education levels [35]. Two-sided tests will be performed for all analyses and the level of significance will be set at *P* < 0.05. Where appropriate, 95% confidence interval will also be reported along with the *P* values.

### Economic analysis

The cost-effectiveness of the ACCM model intervention will be evaluated by comparison of the intervention cost to the expected key outcomes. The following model will be used:

$$ \mathrm{Cost}\kern0.5em \mathrm{Effectiveness}=\left(\frac{Cost_{New}-{Cost}_{Old}}{Effest_{New}-{Effest}_{Old}}\right) $$

### Trial status

We commenced a pilot study in November 2017 to test the implementation of learning modules in two sites outside of the selected sites in the main study. The pilot study will be completed by September 2018. The main study will take 20 months, and is expected to be completed by August 2020.

## Discussion

Previous studies demonstrated that use of the mentor and champion model in the workplace promoted evidence-based practice in acute care hospitals [36–38]. However, there are barriers to implementing this model in nursing homes. First, mentees, who are mainly unlicensed personal care assistants, require the Clinical Mentor to assess their learning needs and initiate learning activities to meet their needs at the point of care. The present project has considered this contextual factor and has allocated eight hours per week for the Clinical Mentor and Site Champions to work with personal care assistants in the education program. Second, nursing homes are viewed as resource-poor settings for conducting education and training for staff [39]. Developing educational resources for staff engaged in the evidence-based quality improvements is imperative. In the present study, the learning modules developed to support the trial will address the education resource issue. The train-the-trainer model and coaching for Site Champions are crucial intervention components that were previously reported in a successful evidence-based quality improvement study [40]. The present study has adapted these components, but emphasizes leadership for Clinical Mentors and Site Champions in the intervention context.

Organizational support through investing time and budget in staff development is viewed as a prerequisite for successful quality improvement [40, 41]. In the present study, the in-kind contributions made by participating nursing homes to support the time the Clinical Mentor and Site Champions spend on the intervention is evidence of organizational support. Policy development to mandate staff education and training is a way to enable and sustain organizational commitment to evidence-based quality improvement in nursing homes in low- and middle-income countries. China is progressing in improving its aged care system which is evidenced by government administered regulations and requisite standards; organizational and governance structures; required staff to resident ratio; and required education and training qualification for staff [42]. All nursing homes are required to meet the standards for nursing homes for accreditation by the National Health and Family Planning Commission of the People’s Republic of China [43]. Nursing homes that have agreed to participate in the study view the ACCM model as an opportunity to establish an organizational structure for sustainable quality improvement and staff development. In the consultation with these participating nursing homes, the management group particularly valued the audit activities embedded in the study, as the audit data provided them with timely and relevant information about quality improvement. The management group also sees this study as an opportunity to empower personal care staff to participate in decision-making processes in quality improvement in which their perspectives about change will be heard.

One of the challenges identified in the previous study was the resistance to the ACCM program from management and staff because staffing levels were low, and mentoring activities and changes in practice were viewed as additional workload for staff [10]. We have considered this challenge and will streamline mentoring activities in existing staff training and supervision activities in the workplace with minimal interference to routine care activities based on consultation with management and staff. Clinical Mentors and Site Champions will implement learning activities at opportunistic moments and at the point of care [39]. The packet workbook for staff allows them to access learning resources to work with the Clinical Mentor in learning opportunities including bedside coaching in residents’ rooms when appropriate. This was tested in a previous mentor and champion model where mentoring activities provided staff with timely support in problem solving, which was successful in reducing their stress levels and improving their job satisfaction and staff retention [24].

The learning modules for staff are mainly based on short case studies that have been collected from the nursing home environment and are relevant to staff members’ daily care activities. These authentic case studies have been adapted to simulate problems and provide solutions in quality improvement. These case studies also enable staff to reflect on their situations and empower them to consider different approaches to problem solving. Case studies also encourage staff to build teamwork, collaboration and adjust their actions in the team in order to generate collective action for better outcomes for residents.

This study comes at an opportune time to introduce the ACCM Model of Change to implement evidence-based quality improvements in nursing homes in China where suboptimal care services are reported and concerns have been raised by the public [6]. The Central Government and the Provincial Government are taking action to address quality of care evidenced by policies, standards and regulations for nursing homes [42]. Findings from this study will inform evidence-based policy to govern quality and safety in nursing homes. Findings will also support policy and resource development to ensure adequate staffing and RNs’ leadership in evidence-based practice and quality improvement in nursing homes.

## Additional file

Additional file 1: SPIRIT 2013 Checklist. (DOC 138 kb)

## Abbreviations

ACCM: Aged Care Clinical Mentor
ADQ: Approaches to Dementia Questionnaire
PCAs: Personal Care Assistants
RCT: Randomized controlled trial
RNs: Registered Nurses
